# Neurophysiological mechanisms of breathing-based well-being practices: a narrative review for clinical application

**DOI:** 10.3389/fpsyt.2026.1774490

**Published:** 2026-07-08

**Authors:** Pravesh Sharma, Vaishnavi Veerareddy, Isu Hong, Marin Nycklemoe, Mahathi Kandimalla, Vamika Sharma, Tiffany L. Kung, Rowa Osman, Seokbeen Lim, Karunya K. Kandimalla, Maria I. Lapid, Paul H. Min

**Affiliations:** 1 Department of Psychiatry and Psychology, Mayo Clinic Health System, Eau Claire, WI, United States; 2 Department of Pharmaceutics and Brain Barriers Research Center, University of Minnesota, College of Pharmacy, Minneapolis, MN, United States; 3 Department of Radiology, Mayo Clinic, Rochester, MN, United States; 4 Department of Psychiatry and Psychology, Mayo Clinic, Rochester, MN, United States; 5 Department of Neurologic Surgery, Mayo Clinic, Rochester, MN, United States; 6 Department of Physiology and Biomedical Engineering, Mayo Clinic, Rochester, MN, United States

**Keywords:** anxiety and stress, breathing, cardiac vagal (parasympathetic) nerve activity, neurophysiological, wellbeing

## Abstract

Breathing is a physiological process that extends beyond gas exchange, linking neural, cardiovascular, glymphatic, and autonomic systems. This narrative review summarizes how breathing-based practices modulate physiological processes in the body and brain, emphasizing their applicability in clinical care. Regulating the pace and depth of breathing, particularly with relaxed exhalation, may engage vagal pathways that lower heart rate and blood pressure and promote a shift toward increased parasympathetic tone relative to sympathetic activity. Neuroimaging studies show that controlled breathing reduces activity in stress-related brain regions while enhancing networks supporting calm attention and emotional steadiness. Changes in breathing depth also influence cerebrospinal fluid movement, though direct evidence of waste clearance in humans is lacking. This review identifies populations requiring caution, including individuals with panic symptoms, significant cardiopulmonary disease, or pregnancy. Clinicians should distinguish between breathing-only and multimodal protocols when interpreting research findings, as combined interventions preclude attribution of benefit to breathing alone. Study design quality, particularly the use of active rather than waitlist controls, should inform how effect sizes are interpreted. Breathing practices are not a replacement for medical treatment but may represent accessible, low-cost adjuncts for stress reduction and cardiovascular regulation.

## Introduction

Structured breathing practices have long been considered broadly accessible tools for modulating physiological and psychological states (1). However, tolerability varies with technique and population. For example, patients with anxiety sensitivity, panic disorder, significant cardiopulmonary disease or autonomic vulnerability may require tailoring and close monitoring (*discussed later*). Their popularity has surged in recent years, driven by public interest and social media dissemination, as well as other community platforms. Clinical researchers have taken a keen interest in studying breathing techniques as non-pharmacological interventions for pain, cognition, psychological, and cardiovascular symptoms (2).

Breathing-based well-being practices are observed across all age groups and rooted in diverse cultural traditions. Despite their cultural heterogeneity, these practices exhibit conceptual convergence, emphasizing the regulation of breath as a means to influence mind–body integration (**Table 1**). Although these traditions share an emphasis on breath regulation, this conceptual convergence should not be taken to imply identical mechanisms or clinical effects. Practices may differ substantially in technique, intention, posture, attentional focus, breath retention, ventilation pattern, and cultural meaning, and their effects require independent empirical investigation. Representative examples include, but are not limited to, Prāṇāyāma in India, Qigong in China, sūsoku-kan in Japanese Zen traditions, and Danjeon Breathing in Korea which is conceptually related to the Chinese concept of Dantian (3, 4). Other culturally grounded traditions emphasize Tummo in Tibetan Vajrayāna meditation (5), Ānāpānasati in early Buddhism, Sufi breath practices, and indigenous rituals worldwide (2, 6).

**Table 1:** Proposed physiological and clinical distinctions across common structured breathing patterns.

Feature	Deep breathing	Slow breathing	4-7-8 Breathing (common wellness practice)	Deep slow breathing (DSB)
Tidal Volume	Increased (diaphragmatic descent and full lung recruitment)	Increased (compensatory rise to maintain ventilation at slow rate)	Mildly increased	Increased (combines large volume and slow pace)
Respiratory Rate	Normal or slightly reduced (10–12 breaths/min)	4–6 breaths/min	3–4 breaths/min (one full 16 sec cycle)	4–7 breaths/min
Exhalation Emphasis	Not emphasized	Equal or slightly longer exhalation	Prolonged exhalation (8 sec)	Prolonged exhalation common
Breath Hold	No	No	Yes (after inhale)	Sometimes (brief post-exhalation rest)
CSF Flow Impact	Increased via large thoracic pressure swings	Present but modest compared to deep breathing patterns	Unknown	Increased via combined volume and timing effects
Autonomic Modulation	Moderate cardiac parasympathetic effect; increases vagally mediated HRV slightly if rate unchanged	Increased cardiac vagal activity; improves vagally mediated HRV and baroreflex sensitivity	Increased parasympathetic activity; decreased sympathetic activity	Increased vagally mediated HRV and baroreflex coherence
Mechanism	Diaphragmatic pressure changes; mild baroreflex stimulation	Baroreflex entrainment; vagal afferent activation	Mild CO_2_ accumulation during brief breath hold; vagal activation; sympathetic inhibition	Combined effects: baroreflex resonance; vagal stimulation; CSF flow; CO_2_ retention
Clinical Use	Primarily used as preparation for other techniques or combined with mindfulness; hypertension	Stress; hypertension; anxiety	Acute anxiety; calming	Anxiety; depression; stress; autonomic dysregulation
Session Duration	5–10 min	10–20 min	10–15 min	10–20 min
Recommended Frequency	As needed	Once or twice daily	Once or twice daily	Once or twice daily
Supervision Needed	No for healthy adults	No for healthy adults	Initial guidance recommended	No for healthy adults; caution advised in anxiety sensitivity and cardiopulmonary disease

CSF, cerebrospinal fluid; HRV, heart rate variability; DSB, deep slow breathing; breaths/min, breaths per minute. Mechanisms listed reflect a range of evidential support. Some pathways are directly established in human studies; others are inferred from animal models or indirect human evidence and have not been confirmed in controlled breathing trials.

Traditional practices often combine breathing synchronized with posture, sound (e.g., mantra), and focused attention. Simultaneously, modern practices have gained prominence in recent years, such as the Buteyko Method for respiratory health, Holotropic Breathwork, Rebirthing Breathwork, the Wim Hof Method combining breath with cold exposure, and coherent breathing techniques for stress and cardiovascular regulation (7–9).

These practices are mechanistically heterogeneous across four key dimensions: rate of respiration (slow paced vs normal vs accelerated), depth of breathing (diaphragmatic/high tidal volume vs reduced volume), breath hold patterns (after inhalation vs after exhalation vs absent) and carbon dioxide (CO_2_) retention approaches (hypocapnia, vs. eucapnia, vs. hypercapnia). For example, slow-paced coherent breathing targets baroreflex engagement under eucapnic conditions, Buteyko emphasizes reduced ventilation to promote CO_2_ retention, and Wim Hof-style cyclic hyperventilation produces hypocapnia through accelerated breathing paired with cold exposure. These dimensions drive distinct physiological pathways that are not interchangeable, and understanding them is essential for clinicians selecting or recommending a practice. Comparative effectiveness research is needed to fully characterize the neural and autonomic targets of each pattern for specific clinical indications.

Clinicians increasingly encounter patients who report practicing, or seeking guidance on, various breathing techniques. These practices have become widespread in contemporary contexts such as yoga studios, wellness programs, and community-based breathing groups. However, many clinicians possess limited knowledge and formal training regarding the diverse typologies of structured breathing practices and their underlying neurophysiological correlates. A concise yet evidence-based review is therefore warranted to update busy clinicians about the range of structured breathing practices and their underlying neurophysiological and psychological mechanisms to better inform themselves and their patients. A detailed search strategy and keywords used for this review article are provided as supplement material (S1).

## Translating breathing research into clinical context

Breathing techniques vary in their pacing, airflow pattern, and tolerance goals. Prāṇāyāma centers on nasal or oral patterning with intentional pacing designed to engage parasympathetic regulatory processes. Buteyko breathing emphasizes slow nasal respiration with short, coached breath-holds to increase CO_2_ tolerance. Paced slow–deep breathing (SDB/DSB) combines diaphragmatic movement, larger tidal volumes, and a respiratory rate near six breaths per minute to reduce somatic tension and stabilize blood pressure and heart rhythm signals. Wim Hof-style training, by contrast, uses cyclic hyperventilation to produce brief, controlled drops in oxygen, paired with cold exposure and represents a multimodal protocol rather than an isolated breathing intervention; its outcomes are therefore considered separately below. For clarity, deep breathing, slow-paced breathing, 4-7–8 breathing, and deep slow breathing represent breathing-only protocols, whereas practices such as Sudarshan Kriya Yoga and Wim Hof Method should be considered multicomponent interventions. A systematic review of breathing protocol is outside the scope of this paper. Readers interested in such review, should consult recent meta-analyses (10, 11).

Clinicians should be familiar with the types of breathing protocols that exist. For example, in clinical trials, some protocols were breathing-only, while in others breathing is often combined with other modalities through co-interventions such as cold exposure, progressive muscle relaxation, or structured rehabilitation programs. When breathing is paired with other active components, it is difficult to determine how much of the benefit came from the breathing itself versus the additional intervention or the program as a whole (9, 12, 13). Clinicians should therefore interpret outcomes from these studies as reflecting the combined effect of the broader intervention package rather than breathing alone.

Clinicians should interpret structured breathing studies with care, as studies that compare breathing practices against no treatment or a waitlist tend to make the benefits appear larger than they may be, while studies that compare breathing against another active or structured activity often show smaller or no differences. In addition, it is important to review the type of breathing practice used, including whether it involves high ventilation or controlled hyperventilation versus slow paced breathing, and the specific inhalation to exhalation duration, as these parameters engage distinct physiological mechanisms and may not produce equivalent outcomes.

The largest blinded structured breathing randomized controlled trial (RCT) to date (n=400) found that coherent breathing at about 5.5 breaths per minute produced no greater reduction in stress, anxiety, or depression than paced breathing at 12 breaths per minute, with both groups improving equivalently, suggesting that structured attention to respiration rather than the specific breathing rhythm may be driving outcomes (14). A separate blinded trial of high-ventilation structured breathing with retention (n=200) with an active placebo group showed a statistically significant improvement in stress from pre- to post-intervention, with a significant main effect of time, but no significant difference between groups, suggesting that regular, structured breathing practice of any kind may confer some benefit regardless of the specific technique (15) On the other hand, a 12-week active-comparator trial of slow breathing found no significant differential effect between exhale-greater-than-inhale and equal-ratio conditions, indicating that general slow pacing may matter more than precise ratio parameters, a detail that is frequently emphasized in clinical guidance (16).

This does not negate the clinical value of breathing practices, but it does underscore the importance of design quality when interpreting effect sizes in this literature.

## Physiological foundations and mechanistic distinctiveness of breathing practices

Mindfulness-based interventions are low-cost, non-pharmacological interventions with an established body of mechanistic research. Structured breathing practices share these characteristics but differ in one important respect: they directly manipulate quantifiable respiratory parameters, including breathing rate, tidal volume, inhalation-to-exhalation ratio, and breath-hold duration, as described above. Mindfulness-based programs, by contrast, integrate breath awareness within a broader therapeutic framework that also encompasses attentional regulation, cognitive retraining, affective processing, and somatic awareness. This multicomponent structure makes it methodologically difficult to attribute observed outcomes to any single or combined element (s) of the intervention. This observation is not intended as a criticism of mindfulness-based approaches, which have demonstrated meaningful clinical benefits across a range of conditions. Rather, it underscores the importance of distinguishing the mechanistic pathways through which each modality is expected to produce its effects. Structured breathing lends itself to more targeted mechanistic investigation, while mindfulness-based programs produce their effects through several interacting processes that are harder to separate in a controlled research setting.

Breathing itself has been studied as a physiological process for over a century, with respiration recognized as simultaneously modulating the autonomic nervous system, hemodynamics, and cerebrospinal fluid (CSF) dynamics (17) (**Table 1**). While the basic physiology of breathing is well characterized, the specific mechanisms through which structured breathing practices produce clinical benefits are still being defined. An expanding body of research suggests these practices may contribute positively to mental and physical health across conditions ranging from asthma and hypertension to anxiety and mood disorders (10, 18). However, rigorous trials involving large and diverse samples remain scarce, and many observed benefits have yet to be validated through carefully controlled research.

## Cardiovascular and autonomic aspects of respiratory physiology

The autonomic nervous system (ANS) activity measured by HRV is common biomarkers used to describe the physiological responses to breathing practices (19). Below, we provide a brief description of ANS, and HRV to provide background information, followed by a clinical case illustration.

### Autonomic nervous system

There are two parts to the autonomic nervous system, the sympathetic nervous system (SNS) and the parasympathetic nervous system (PNS). These two systems have reciprocal inhibition to one another, and in some organs, such as the heart, they have complementary double innervation. The SNS activates the body’s *‘fight or flight’* response by increasing heart rate, blood pressure, and breathing rate, while suppressing non-essential processes. The PNS, through the vagus nerve, causes the *‘rest and digest’* state by reducing the heart rate and breathing rate and enhancing digestion (20).

### Heart rate variability

HRV refers to the natural fluctuations in the time interval between consecutive heartbeats and reflects the dynamic interplay between sympathetic and parasympathetic branches of the autonomic nervous system. Higher HRV generally indicates stronger parasympathetic influence and greater flexibility in autonomic regulation, whereas lower HRV is associated with reduced vagal activity, heightened physiological stress, and diminished adaptive capacity. HRV is widely accepted as a non-invasive marker of autonomic control and physical stress (21).

Additionally, inhalation reduces vagal output through cardiovascular regulatory centers, shifting autonomic control toward sympathetic dominance and temporarily increasing heart rate. Exhalation, in contrast, is associated with increased vagal activity, which lowers heart rate. These rhythmic changes in heart rate tied to breathing have traditionally been called respiratory sinus arrhythmia (RSA), a term present in the literature for decades. However, a 2025 international expert recommendation (22) has called for retiring this term, arguing that the word “arrhythmia” carries misleading pathological connotations despite RSA being a normal and healthy phenomenon. The authors propose replacing RSA with respiratory heart rate variability (RespHRV), a term that more accurately reflects what is being measured: the variation in heart rate that is specifically driven by breathing. Importantly, RespHRV encompasses respiratory-related heart rate oscillations in both the low-frequency and high-frequency bands, and its amplitude should not be interpreted as a direct measure of vagal tone. This distinction matters clinically because equating RespHRV with vagal tone risks overinterpreting what is in fact a narrower, breathing-coupled index of cardiac autonomic activity.

It is worth noting that not all breathing-related benefits operate through HRV pathways. Balban and colleagues reported that cyclic breathing (a pattern of two sequential nasal inhalations followed by a prolonged oral exhalation) reduced anxiety and improved mood without producing measurable changes in HRV (23). This finding suggests that breathing practices can engage regulatory mechanisms beyond those captured by standard HRV indices, likely involving pathways related to CO_2_ sensitivity, interoception, or central neural regulation. Clinicians should therefore not rely on HRV change alone as the sole indicator of a meaningful physiological response to breathing practice.

The HRV is commonly assessed using two broad approaches: time-domain and frequency-domain analysis. Time-domain measures, such as the standard deviation of normal-to-normal intervals, represent overall variability in heart rate, while the root mean square of successive differences reflects short-term changes that are largely influenced by vagal (parasympathetic) activity (24). Frequency-domain methods, which break the heart rate signal into its component frequencies, provide additional insight into the balance of autonomic influences. The high-frequency band (HF-HRV), closely aligned with the breathing cycle, is used as an index of cardiac vagal activity, though it should not be equated with overall vagal tone, particularly during controlled breathing where respiratory rate itself directly influences HF power. The low-frequency band (LF-HRV) was once viewed as a marker of sympathetic activity, but accumulating evidence shows that it also contains a substantial parasympathetic component. As a result, the traditional LF/HF ratio is now regarded as an unreliable indicator of autonomic influences, which interact in a complex and context-dependent manner, as sympathetic and parasympathetic activity can vary independently rather than in simple reciprocal opposition (25–27).

Spectral analysis of HRV has important methodological limitations. Frequency band boundaries are fixed by convention, yet respiratory rate directly shifts RespHRV oscillations across LF and HF bands during slow breathing, complicating interpretation. HRV indices also do not provide a direct measure of sympathetic activity. Muscle sympathetic nerve activity (MSNA), measured via microneurography, offers a more direct and robust assessment of sympathetic outflow (28, 29). However, the technical demands of microneurography have limited its inclusion in most breathing intervention studies, representing a methodological gap in the field.

From a clinical standpoint, higher HRV generally indicates greater flexibility and resilience of the autonomic nervous system, while persistently low HRV may point to physiologic stress, fatigue, or reduced parasympathetic modulation (30). Interpretation, however, must always take into account the patient’s overall health, emotional state, and measurement context.

### Clinical case illustrations of autonomic modulation

Consider a middle-aged person recovering from a stressful hospitalization. At rest, their heart rate monitor shows a steady rhythm with little beat-to-beat variation—an indication of low HRV, suggesting limited parasympathetic engagement. After several weeks of guided breathing and gentle physical activity, their HRV recordings show greater fluctuation between beats, especially during slow exhalations. This pattern may reflect increased vagal tone and improved autonomic flexibility, even though their resting heart rate remains unchanged. Clinically, this could be consistent with better stress recovery, improved sleep, and a calmer physiological state, though these subjective improvements cannot be attributed to breathing practice alone without more comprehensive assessment.

### Deep and slow breathing

Two broad categories of breathing practices are commonly described: slow-paced breathing and deep breathing. Slow-paced breathing involves intentionally reducing the respiratory rate, typically to about six breaths per minute, with a balanced or slightly longer exhalation phase (31). In contrast, deep breathing emphasizes larger breath volume through diaphragmatic movement (**Table 1**).

Slow-paced breathing synchronizes respiratory rhythms with baroreflex oscillations around 0.1 Hz, enhancing LF HRV, baroreflex sensitivity, and overall autonomic flexibility (32). When the depth of breathing is added to slow timing, particularly with extended exhalation, the modulation of autonomic activity is further amplified. This deep slow breathing (DSB) pattern has been shown to increase vagally mediated HRV indices, including Root Mean Square of Successive Differences and HF power, under controlled breathing conditions at approximately six breaths per minute. These findings are consistent with enhanced cardiac parasympathetic activity, though as noted above, it should be noted that HF-HRV is directly influenced by respiratory rate itself and should not be interpreted as a direct measure of vagal tone. Within these constraints, DSB-associated increases in vagally mediated HRV have been linked to reductions in perceived anxiety even after brief practice sessions (33).

Aside from its impact on the vagus nerve and HRV, paced breathing (a voluntarily regulated breathing pattern in which inhalation and exhalation are consciously timed) is a potent tool for conditioning the baroreflex system (32). This feedback loop helps maintain cardiovascular stability by responding to moment-to-moment changes in heart rate and vessel tone. Baroreceptors in the carotid sinus (neck) and aortic arch (chest) detect fluctuations in blood pressure and relay this information to the brainstem’s nucleus tractus solitarius. The autonomic system then adjusts cardiac output and blood vessel tone accordingly (34). When the rate of breathing declines to about 0.1 Hz (six breaths per minute), its rhythm resonates with the natural frequency of the baroreflex, promoting optimal baroreflex function and physiological coherence, a synchrony among breath, heart, and vascular rhythms that extends beyond mechanical coupling (35).

Neuroimaging studies demonstrate that the alterations in HRV are accompanied by changes in activity within brain regions such as the insula, anterior cingulate cortex, and brainstem, all of which play integral roles in the perception of internal states and emotion regulation. Thus, breathing influences not just the heart but is also associated with changes in brain regions involved in behavior, cognition, and memory, though the functional significance of these changes requires further investigation (36).

Nevertheless, one should think of HRV as a surrogate marker. Although sensitive, it is nonspecific and refers to the overlapping effects of posture, age, respiratory patterns, and stress. A broader understanding of structured breathing practices will likely emerge from the integration of multimodal strategies that integrate HRV with indicators of neural activity and immune response, thereby providing a more comprehensive understanding of how breathing influences the brain and body.

It is important to distinguish between three timeframes of evidence. First, acute effects refer to physiological changes occurring during or immediately after a single breathing session, such as increases in vagally mediated HRV and reductions in anxiety. Second, effects of sustained practice refer to adaptations that develop over weeks to months of regular breathing training, such as persistent improvements in baroreflex sensitivity and resting autonomic tone. Third, durability of effects refers to whether benefits persist after a breathing program ends — an area that remains largely understudied. Evidence for sustained practice effects exists; for example, three months of regular slow breathing produced lasting parasympathetic shifts in healthy adults (37). These effects depend on protocol parameters including breathing rate, session duration, and inhalation-to-exhalation ratio, as well as inter-subject variability and the HRV analysis methods employed.

To apply this distinction concretely, acute mechanistic evidence includes single-session observations such as increases in vagally mediated HRV, transient CSF displacement, BOLD signal changes in autonomic brain regions including the medulla, hippocampus, insula, and anterior cingulate cortex, and immediate reductions in self-reported anxiety. These findings establish physiological plausibility but do not, by themselves, support broader clinical recommendations. Short-term intervention evidence includes adaptations observed over weeks of regular practice, such as sustained improvements in baroreflex sensitivity, resting autonomic tone, and reductions in circulating Interleukin-6 (IL-6) in controlled trials. Long-term and durable clinical evidence, that is, whether benefits persist after a breathing program ends, remains the least studied timeframe, and rigorous longitudinal follow-up data are currently lacking. Clinicians should therefore calibrate their recommendations to the appropriate tier of evidence and interpret effect sizes accordingly, particularly when studies rely on waitlist rather than active comparators.

Clinically, this means that a single session may produce measurable acute physiological changes, particularly relevant to anxiety and autonomic dysregulation, but sustained benefits are more likely to require regular practice over several weeks, with protocol parameters tailored to the individual patient. Standardized protocols and longer-term follow-up are therefore needed in both research and clinical practice to clarify optimal dosing and the durability of effects across different patient populations.

### Clinical case illustration

A patient with mild-moderate anxiety begins practicing DSB at about six breaths per minute, emphasizing a gentle, extended exhalation. During each session, their heart rate rises slightly with inhalation and falls with exhalation, reflecting stronger cardiac vagal activity. After several days of practice, they report feeling calmer and less reactive to daily stressors, an effect that could be consistent with improved baroreflex function, higher HRV, and greater autonomic flexibility, and may correspond to breathing-related changes in autonomic regulation, though confirmation requires more comprehensive assessment.

### Brain correlates of breathing

#### Cerebrovascular reactivity

Cerebrovascular reactivity (CVR) is important in reflecting brain cerebrovascular changes related to breathing. CVR is the ability of cerebral blood vessels to regulate cerebral blood flow (CBF) through dilation or constriction by responding to vasoactive factors (38, 39). CVR is important in providing information of vascular functions, as well as localized cerebrovascular changes (39, 40). It is also used as a biomarker for pathologies where vasculature is compromised for a variety of conditions. It may also be used to look at cerebral spatiotemporal patterns, which may reflect regional variation in the vasculature (38).

When the carbon dioxide (CO_2)_ concentration in the blood and CSF is increased, the CO_2_ will react with water (H_2_O) to form carbonic acid (H_2_CO_3_). After formation, it then dissociates into hydrogen (H^+^) and bicarbonate ions (HCO_3_^-^) ions. This reaction maintains the pH of the blood. When the H^+^ concentration in the blood increases, the pH of the blood is lowered. A lower blood pH stimulates chemical chemoreceptors, inducing vasodilation to then regulate blood pH (39).

Higher CO_2_ levels cause hyperpolarization in endothelial cells, where the electrical signal is then transmitted to smooth muscle cells via gap junctions. This results in the membrane potential becoming more negative, activating potassium (K^+^) channels. Voltage-dependent calcium (Ca^2+^) channels are inhibited, which reduces the intracellular Ca^2+^ concentration. Due to this reduction, vasodilation occurs, resulting in smooth muscle relaxation. Moreover, CO_2_ and pH changes activate nitric oxide synthase in endothelial cells. This increases the production of nitric oxide, which then diffuses into smooth muscle cells and results in relaxation (39).

CVR is measured by applying a challenge to cerebral vasculature, reflecting a change in CBF. The most common challenge is by inducing hypercapnia through the inhalation of hypercapnic gas, such as 5% CO_2_. This increase in blood CO_2_ concentration leads to vasodilation and increased CBF. While this occurs, blood oxygen level-dependent (BOLD) functional MRI images are captured. This method of measurement is thought to reflect CBF changes, as well as quantify CVR (38, 40).

The CO2 mediated vascular mechanisms are directly engaged by voluntary breathing practices. When respiratory rate is reduced during slow paced breathing, tidal volume increases compensatorily to maintain ventilation, which together produce mild CO2 retention and promote cerebral vasodilation through the mechanism described above. Slow paced breathing at approximately 6 breaths per minute may improve the efficiency of alveolar ventilation, reduce dead space, and enhance cardiorespiratory coupling under typical resting conditions, effects that are thought to extend to cerebrovascular regulation through CO_2_-driven changes in cerebral blood flow (1).

#### Cerebrospinal fluid dynamics during breathing practice

CSF and interstitial fluid (ISF) play a crucial role in maintaining CNS homeostasis by facilitating the distribution of nutrients and hormones and promoting the clearance of metabolites and toxic Aβ peptides from the brain parenchyma (41). These processes are accomplished by the essential mixing of CSF from the subarachnoid space with ISF, which is primarily mediated by cerebral arterial pulsatility (42). Disruption of these important functions is implicated in the pathophysiology of various neurodegenerative and neuroinflammatory conditions.

The glymphatic pathway orchestrates the distribution and clearance systems mediated by the CSF. Initially characterized by Maiken Nedergaard’s group in rodent models (43), this pathway enables CSF to flow from the subarachnoid space into the brain parenchyma via perivascular spaces. Human evidence for a comparable pathway relies primarily on indirect measures; recent studies, conducted using intrathecal contrast-enhanced MRI, have also confirmed the existence of a similar CSF flow mechanism in humans (44), though direct visualization as achieved in rodent models is not feasible in living humans. Whether breathing practices specifically enhance glymphatic clearance in humans to the same degree observed in animal models remains to be established.

Our understanding of what drives CSF and glymphatic flow continues to evolve. The CSF flow is primarily controlled by alterations in the central nervous system (CNS) vascular bed, including cardiac pulsations and respiration, and secondarily by changes in body position and the cough reflex. Historically, cardiac pulsations were considered the primary force regulating CSF dynamics (45). However, real-time MRI studies in healthy individuals have demonstrated that respiration is one of the predominant drivers of CSF movement in humans (46, 47). Consistently, Yildiz et al. have shown that yogic breathing impacts pulsatile CSF dynamics (48). Although heart-driven CSF pulsations are still observable during breathing, especially in the cervical spine near the heart, this baseline rhythm is now considered to be superimposed by the significantly greater amplitude of the flow component generated by respiration (49). In line with this, recent real-time velocity-encoded MRI data demonstrate that, in breathing-trained participants, even during their regular breathing, CSF displacement and net flow at the foramen magnum and lateral ventricle are enhanced and are strongly associated with diaphragm movement, supporting respiration as a modifiable driver of CSF dynamics in the awake state (47).

**Figure 1:**
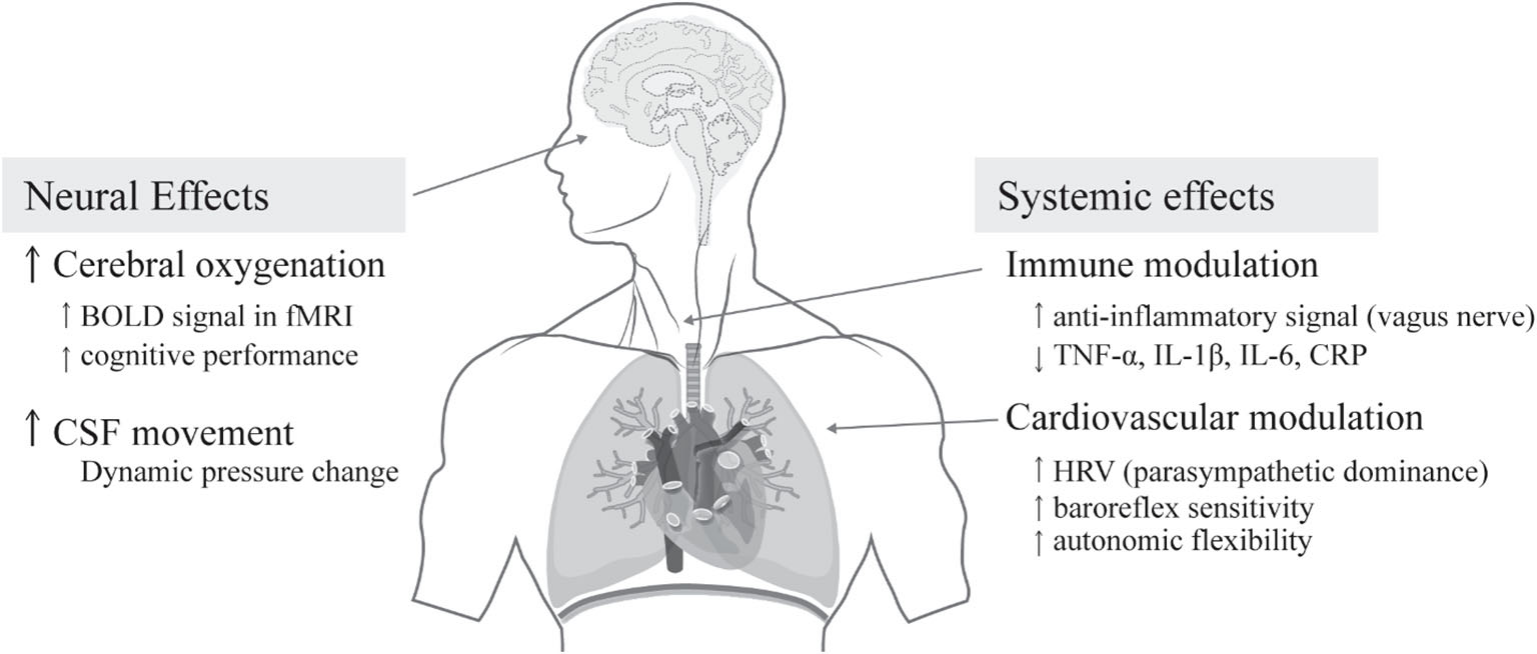
Neurophysiological mechanisms of breathing-based practices. Summary of the key neural and systemic pathways influenced by structured breathing practices. Deep breathing has been associated with enhancement of cerebral oxygenation and may influence cognitive performance, increases cerebrospinal fluid (CSF) flow though the functional significance of this for waste clearance in humans is not yet established, may modulate immune responses through vagal activity (reducing pro-inflammatory cytokines), and may promote cardiovascular regulation by increasing heart rate variability (HRV) and baroreflex sensitivity. Together, these integrated mechanisms may position structured breathing practices as accessible tools for the modulation of brain, cardiovascular, and immune function, supporting emotional and physical well-being. Pathways depicted represent varying levels of evidential support. Some mechanisms are directly established in human physiological studies; others are inferred from animal models or theoretical frameworks and remain to be confirmed in controlled human breathwork trials.

The respiratory mechanism driven by deep diaphragmatic breathing causes significant shifts in intrathoracic and intra-abdominal pressures (**Figure 1**). During inspiration, decreased intrathoracic pressure promotes venous drainage from the brain and neck, leading to a transient reduction in cranial blood volume (50). According to the Monro–Kellie doctrine, this drop is compensated by an upward movement of CSF from the lumbar spine through the spinal canal into the cranial vault to maintain intracranial pressure equilibrium (49, 51). During expiration, a partial downward flow of CSF occurs, creating a rhythmic and bidirectional current (52).

These findings suggest that respiration influences CSF dynamics, and while CSF and glymphatic pathways are implicated in solute clearance in animal models, whether breathing practices meaningfully enhance waste clearance or confer neuroprotection in humans remains to be established. Further, slow-paced breathing has been shown to reduce plasma amyloid-β levels, potentially via noradrenergic modulation in one study but replication is needed (53).

#### Impact of breathing on cerebral blood flow, brain oxygenation, and brain activity

Breathing profoundly impacts brain function by regulating CBF through its influence on the vascular endothelium. Breathing may influence the neurovascular coupling, the mechanism that links neuronal activity to local changes in blood flow, by directly influencing blood CO_2_ levels (54). The brain relies on rapid and predictable vascular responses to CO_2_ fluctuations generated by even small breathing changes that occur during everyday actions, such as speaking or sighing. The vascular endothelium regulates cerebrovascular reactivity, and its function is disrupted by endothelial dysfunction, which impairs the brain’s ability to regulate blood flow in response to CO_2._ Endothelial dysfunction is prevalent in conditions such as metabolic syndrome, cerebrovascular pathology, and Alzheimer’s disease (55).

The neuronal activity in a specific brain region requires more oxygen, resulting in increased blood flow to that region. This is reflected as an enhanced BOLD signal in Functional Magnetic Resonance Imaging (fMRI) and is widely recognized as an indirect measure of brain activity. Using fMRI, Critchley et al. found that slow breathing increased the BOLD signal in brainstem and subcortical regions, including the dorsal pons, periaqueductal grey matter (PAG), thalamus, and hippocampus, as well as sensorimotor cortical areas, notably under hypoxic challenge conditions. Across conditions, medullary and hippocampal activity correlated positively with HRV (56). It should be noted that these BOLD findings were observed under hypoxic challenge conditions rather than standard resting breathing, and while end-tidal CO_2_ was monitored and included as a covariate in the analyses, the authors acknowledge that non-neural contributions to the BOLD signal cannot be fully excluded. Observed signal changes should therefore not be interpreted as direct indices of improved neural function. In contrast, HRV was negatively correlated with activity in the anterior insula, dorsomedial prefrontal cortex, and left occipital cortex. The findings provide neurobiological support for slow breathing practices, suggesting that they engage brainstem and subcortical circuits involved in autonomic regulation, while regions associated with interoceptive monitoring show an inverse relationship with vagal activity (56). Clinically, this implies that the benefits of slow breathing extend beyond peripheral reflexes, reflecting a central pattern consistent with reduced cortical arousal and enhanced subcortical autonomic control.

Additionally, slow and controlled breathing has been associated with an increase in cerebral oxygen levels and stabilization of brain activity patterns, which could improve emotional regulation and cognitive performance (56). It is also shown that breathing may influence neural oscillations by increasing alpha waves (8–13 Hz), which are associated with relaxed, focused attention and emotional regulation, and decreasing theta waves (4–8 Hz), which are associated with reduced rumination and mental noise (57).

#### Vagal nerve-mediated immune modulation through breath

The cholinergic anti-inflammatory reflex is a neurologic response whereby the vagus nerve perceives peripheral inflammation and signals the brain to modulate the immune response (**Figure 1**). Upon stimulation, the efferent vagus signals the spleen and other immune organs to suppress the production of pro-inflammatory cytokines such as Tumor Necrosis Factor-alpha (TNF-α), Interleukin-1 beta (IL-1β), and IL-6. This is followed by the release of acetylcholine, which activates the α7 nicotinic acetylcholine receptors (α7nAChRs) on macrophages and decreases their inflammatory output (58). Existing evidence suggests that slow-paced breathing and other controlled breathing methods may increase cardiac vagal activity, as reflected by vagally mediated HRV; however, HRV changes alone do not demonstrate activation of the cholinergic anti-inflammatory reflex. Neuroimaging and HRV research show that slow breathing increases cardiac vagal activity as reflected by HRV indices, which may engage broader efferent vagal pathways including the brainstem–vagus–spleen axis, though HRV alone cannot confirm activity in these peripheral vagal branches (59).

An RCT in patients with moderate COVID-19 pneumonia provides direct clinical support, demonstrating that slow-paced breathing at six breaths per minute significantly reduced circulating IL-6 levels compared to controls, with a small to medium effect size and no relevant side effects, though the authors note that larger trials are needed to confirm these findings (60). Early clinical trials (e.g., yoga, meditation, and broader structured breathing interventions) observed decreases in circulating levels of IL-6 and C-reactive protein, findings supported by a recent meta-analysis of 89 randomized controlled trials of mind-body interventions (61). However, those interventions were multicomponent in nature, and the specific contribution of breathing cannot be isolated from those findings. Readers should therefore interpret these immune findings as reflecting the broader mind-body intervention rather than breathing alone.

This reflex is also being targeted in vagus nerve stimulation (VNS) trials for rheumatoid arthritis, inflammatory bowel disease, and long COVID with pilot studies reporting symptom relief and immune recalibration (62). While VNS targets this circuit directly, whether slow-paced breathing engages the same efferent pathways to a clinically meaningful degree remains to be established. As auricular and other non-invasive VNS approaches remain under investigation, behavioral interventions such as structured breath training represent a plausible and low-cost candidates for modulating cholinergic anti-inflammatory signaling, though direct evidence in humans is currently limited to preliminary findings (62).

### Adverse effects

Although breathing practices provide significant benefits, they may also entail adverse effects and may not be suitable for all patient populations. For example, slow and deep breathing may induce acute dizziness and lightheadedness due to respiratory alkalosis and the associated reduction in CBF (63). These effects are usually transient but can be distressing in individuals with anxiety (64).

In some individuals, breathing interventions may exacerbate anxiety rather than alleviate it. Alterations in autonomic activity and interoceptive sensations, such as palpitations or tingling, can mimic the somatic cues of panic attacks (65). Moreover, individuals with acute stress disorders may re-experience intrusive traumatic memories during hyperventilation, highlighting the need for careful screening and supervision in patients with psychiatric histories when applying these practices (66).

Hyperventilation can disrupt normal self-monitoring and bodily awareness, leading some individuals to experience altered states of consciousness, including feelings of detachment or loss of self-boundaries (67). Although rare, breathing practices have also been reported to trigger perceptual disturbances, including visual and auditory hallucinations, likely due to changes in cerebral excitability and oxygen–carbon dioxide balance (68). Therefore, proper professional training is recommended, along with the use of structured and systematic breathing practices, to minimize risks and ensure safe application.

Contraindications for structured breathing practices extend across both psychiatric and medical domains. Patients with epilepsy, panic disorder, or psychotic disorder demonstrate increased vulnerability to respiratory manipulations and may exhibit exaggerated neurophysiological and metabolic responses (69). Likewise, patients with significant cardiovascular (e.g., malignant hypertension, arrhythmias, ischemic heart disease, aneurysms, heart failure), cerebrovascular (e.g., ischemic or hemorrhagic disease, cerebral aneurysms), or respiratory conditions (e.g., Chronic Obstructive Pulmonary Disease) should engage in these practices under close medical supervision, as transient but marked shifts in blood pressure, vasoconstriction, and cerebral blood flow may pose undue risks. Pregnant individuals and those with pheochromocytoma should avoid high-ventilation and breath-hold protocols; gentle slow-paced breathing may be appropriate under medical guidance (67).

A practical approach to safety requires distinguishing between technique types and patient populations. Based on the available evidence, we propose the following approach. Gentle slow-paced breathing at six breaths per minute with natural tidal volume is considered low risk for most healthy adults and can generally be introduced without supervision (1). Practices requiring caution or professional guidance include breath retention protocols, forceful pranayama such as Kapalabhati (67) and resonance frequency biofeedback in patients with pacemakers or conditions producing metabolic acidosis (32). High-ventilation and hyperventilation-based practices should be avoided in patients with epilepsy (69), panic disorder, or significant cardiopulmonary compromise (67). Patients with acute stress disorders may re-experience intrusive traumatic memories during hyperventilation and require careful screening and supervision (66). Breath-hold protocols carry additional cardiovascular risk and should be approached with caution in patients with cardiovascular disease and hypertension (67). Cold-exposure protocols, as used in the Wim Hof method, represent a separate multicomponent risk category beyond breathing alone (67). Pheochromocytoma represents an absolute contraindication due to vasopressor crisis risk (67). Although rare, perceptual disturbances including hallucinations have been reported during hyperventilation (68). Red-flag symptoms requiring immediate discontinuation include pre-syncope, chest pain, palpitations, and new neurological symptoms during practice (67).

### Current research gaps

There remains a significant gap in breathing science literature regarding a clear dose–response relationship. Some studies have demonstrated that longer durations of deep breathing produced the most reliable improvements in HRV. However, most protocols still rely on arbitrarily chosen session lengths without systematic testing of optimal durations (70). Additionally, the dose-response effects of breathing training have not been systematically characterized, underscoring the need for more rigorous, multi-dose trials (31).

There is a growing need to evaluate the wide range of existing breathing practices to identify the most consistent and effective approaches. Many techniques show promising physiological and psychological effects, but meaningful comparison is limited by the lack of standardized research methodology. Developing uniform frameworks for assessing these practices would allow clearer determination of which techniques offer the most reliable therapeutic benefit.

Additionally, most experimental paradigms rely on laboratory-controlled, short-term protocols, offering limited insight into the effects of prolonged or stage-based training on brain function and behavior. Evidence from Sudarshan Kriya Yoga in post-traumatic stress disorder patients and in physicians demonstrated that multi-stage and long-term breathing practices produced benefits lasting months to a year, far exceeding acute effects typically captured in laboratory studies (71).

Taken together, these gaps highlight the need for systematic investigation of dose–response effects, standardized research frameworks, and long-term mechanisms in breathing science. Many established breathing approaches use a structured, stage-based progression that gradually increases complexity and engagement. Such models offer a useful framework for examining how progressive training influences physiological and psychological outcomes over extended periods. Incorporating these structured approaches into research may clarify optimal dosing, reveal mechanisms that support sustained benefits, and guide development of more effective clinical protocols.

### Implications for clinical practice, research, and patient engagement

Expanding this knowledge could have meaningful practical applications. At the same time, clinicians should be aware that current comparative evidence does not firmly establish that any one breathing technique outperforms another for most psychiatric or cardiovascular outcomes, and that structured attention to breathing itself may account for a substantial portion of observed benefits. In clinical practice, clinicians can use **Table 2** as a concise reference guide when explaining the neurophysiological mechanisms underlying structured breathing practices to patients, helping translate complex concepts such as cardiac vagal nerve activation, HRV, baroreflex function, and brain–body regulation into accessible and clinically relevant explanations. Breath regulation strategies can then be applied more precisely and effectively as part of integrative care for conditions such as anxiety, insomnia, or cardiovascular dysregulation. For researchers, identifying these underlying neural and autonomic pathways can help shape more precise hypotheses and measurement strategies. From a patient perspective, having a clear, mechanism-based explanation grounded in **Table 2** may enhance understanding, credibility, and adherence by linking subjective benefits to identifiable biological processes. Overall, a better understanding of the mechanisms summarized in this paper can advance the field toward evidence-based, individualized use of structured breathing practices and guide the design of future studies using neuroimaging, electrophysiology, and biomarkers to test causal models.

**Table 2:** A quick reference when explaining the proposed mechanisms of structured breathing practices to patients.

Neurophysiological mechanism	Potential physiological role
Cardiac Vagal Modulation	Increase cardiac parasympathetic activity, slowing heart rate and reducing sympathoadrenal activation
Heart Rate Variability	Reflects balance between stress and calm systems, improves resilience.
Baroreflex Training	Stabilizes blood pressure and heart rhythm with slow, steady breathing.
Brainstem Modulation	Regulates breathing rhythm and calm-alertness balance.
Insula & ACC Engagement	Supports emotional regulation and awareness of body state.
Neural Oscillation Changes	Promotes calm and focus by increasing alpha brain waves.
Cerebrospinal Fluid Flow	Enhances CSF fluid circulation; direct evidence of waste clearance in humans is not yet established.
Anti-inflammatory Reflex	May reduce inflammation via vagus nerve-mediated immune modulation.

## Conclusion

Structured breathing practices represent a low-cost intervention with measurable physiological effects that can be used in clinical settings for patients with stress-related, emotional, or cardiovascular dysregulation. However, comparative trial evidence does not yet establish clear superiority of specific breathing ratios or named techniques for most clinical outcomes, and clinicians should interpret technique-specific claims accordingly. These practices increase vagally mediated HRV, consistent with enhanced cardiac parasympathetic activity, though broader vagal pathways including those involved in immune modulation involve distinct efferent circuits that cannot be directly inferred from HRV alone. Breathing practice also influences baroreflex function, helping stabilize blood pressure and heart rate, and engages neural circuits involved in self-awareness and affect regulation, including the anterior cingulate cortex and insula. In addition, breathing may enhance cerebrospinal fluid circulation, has been associated with changes in cerebrovascular parameters that may influence oxygen delivery, may reduce the accumulation of metabolic waste, and may activate anti-inflammatory pathways through the cholinergic reflex. It should be noted that evidence cited for immune modulation and some psychological outcomes derives from multicomponent interventions, and attribution of benefit to breathing alone is not possible in those cases. Together, these convergent mechanisms suggest that breathing-based interventions may serve as a valuable complement to standard therapies for conditions such as anxiety, hypertension, and chronic inflammation, with growing neurobiological evidence supporting their broader integration into clinical practice.
