# Supplementary material for: Neurophysiological mechanisms of breathing-based well-being practices: a narrative review for clinical application
Source: Front Psychiatry. 2026 Jul 8;17:1774490. doi: 10.3389/fpsyt.2026.1774490 (PMC13389407; doi:10.3389/fpsyt.2026.1774490)
Supplement: Supplementary file 1 [file Table1.docx]

Neurophysiological Mechanisms of Breathing-Based Well-Being Practices: A Narrative Review for Clinical Application

Search strategy and Keywords:

To keep the search comprehensive and timely, the review encompassed publications through the current date and incorporated recent research across multiple domains. The keywords used systematically for this exploration are detailed by topic. For comparative approaches and clinical outcomes across structured breathing trials, keywords include breathing practices and cultural context; pranayama clinical trial; qigong breathing HRV; standardized breathing protocols; mind-body interventions and cultural tailoring; culturally adapted interventions and mind-body. For exploring cerebrovascular reactivity, keywords used were: Cerebrovascular reactivity and Clinical Symptoms; Cerebrovascular reactivity; cerebrovascular reactivity mechanism; Cerebrovascular reactivity and stroke; Cerebrovascular reactivity and clinical application. Breathing methods were investigated using the following keywords: Pranayama breathing, clinical studies, clinical outcomes; pranayama breathing; Wim Hoff breathing and clinical outcomes; Wim Hoff method, clinical applications, and stroke; deep slow breathing; deep slow breathing abd clinical applications; Buteyko method; Buteyko method and clinical application. For breathing methods relevant adverse effects, the keywords include: Breathwork review; hyperventilation, panic disorder, stress disorder, and hallucination; breathwork, panic disorder, and stress disorder. For the nervous system, keywords include: Autonomic nervous system, vagus nerve stimulation, cholinergic anti-inflammatory reflex, vagus–spleen axis.
